# Supplementary material for: Distinct functions of POT1 proteins contribute to the regulation of telomerase recruitment to telomeres
Source: Nat Commun. 2021 Sep 17;12:5514. doi: 10.1038/s41467-021-25799-7 (PMC8448735; doi:10.1038/s41467-021-25799-7)
Supplement: Supplementary file 1 — Supplementary Information [file 41467_2021_25799_MOESM1_ESM.pdf]

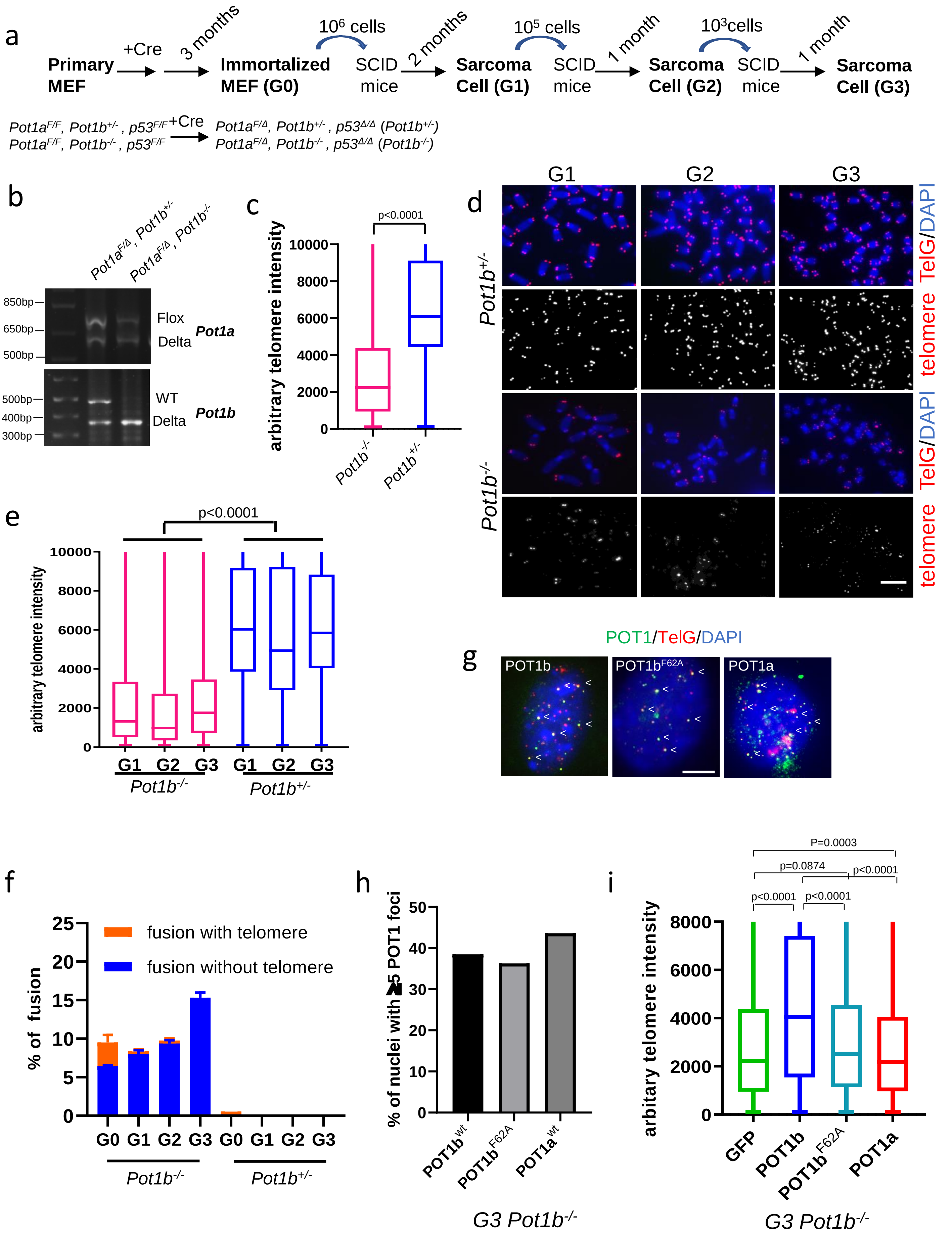

Supplementary figure 1

Supplementary figure 1. Generation and characterization of SCID tumor cell lines. a. Schematic of SCID sarcoma cell lines generated by repeated injections of immortalized MEFs into SCID mouse. **b.** Genotypes of *Pot1a*$^{F/\Delta}$*; Pot1b*$^{+/-}$*; p53*$^{\Delta/\Delta}$ and *Pot1a*$^{F/\Delta}$*; Pot1b*$^{-/-}$*; p53*$^{\Delta/\Delta}$ G0 MEFs. **c.** Quantification of arbitrary telomere intensity of *G3 Pot1b*$^{+/-}$ and *G3 Pot1b*$^{-/-}$ cells (related to **Figure 1B**). Box show the median and interquartile range (25%-75%) from three independent experiments. p-values were generated from unpaired student's t-test. **d**. Telomere PNA-FISH analysis of three generations of *Pot1b*$^{+/-}$ and *Pot1b*$^{-/-}$ SCID tumor cell lines. Scale bar: 5µm**. e.** Quantification of relative telomere intensity in (**d**) by Q-FISH analysis. Box show the median and interquartile range (25%-75%) from three independent experiments. p-values were generated from unpaired student's t-test. **f.** Quantification of telomere fusions in (**d**). Percentage of chromosome fusions without (blue) and with (orange) telomeres at sites of fusions observed in *Pot1b*$^{-/-}$ and *Pot1b*$^{+/-}$ tumors of the indicated generations. Data show the mean ± s.d. from two independent experiments. A minimum of 5000 chromosome ends were counted for each sample in each experiment. **g.** Co-localization (white arrows) of POT1b^{WT}, POT1b^{F62A} or POT1a (green) on telomeres (red) in *G3 Pot1b*$^{-/-}$ sarcomas. Scale bar: 5µm**. h**. Quantification of POT1b^{WT}, POT1b^{F62A} or POT1a co-localized on telomere in (**g**). **i**. Quantification of telomere intensity in *G3 Pot1b*$^{-/-}$ reconstituted with indicated DNA constructs (related to **Figure 1e**). Data show the median with interquartile range (25% - 75%) from three independent experiments. p-values are shown and generated from one-way ANOVA analysis followed by Tukey's multiple comparison.

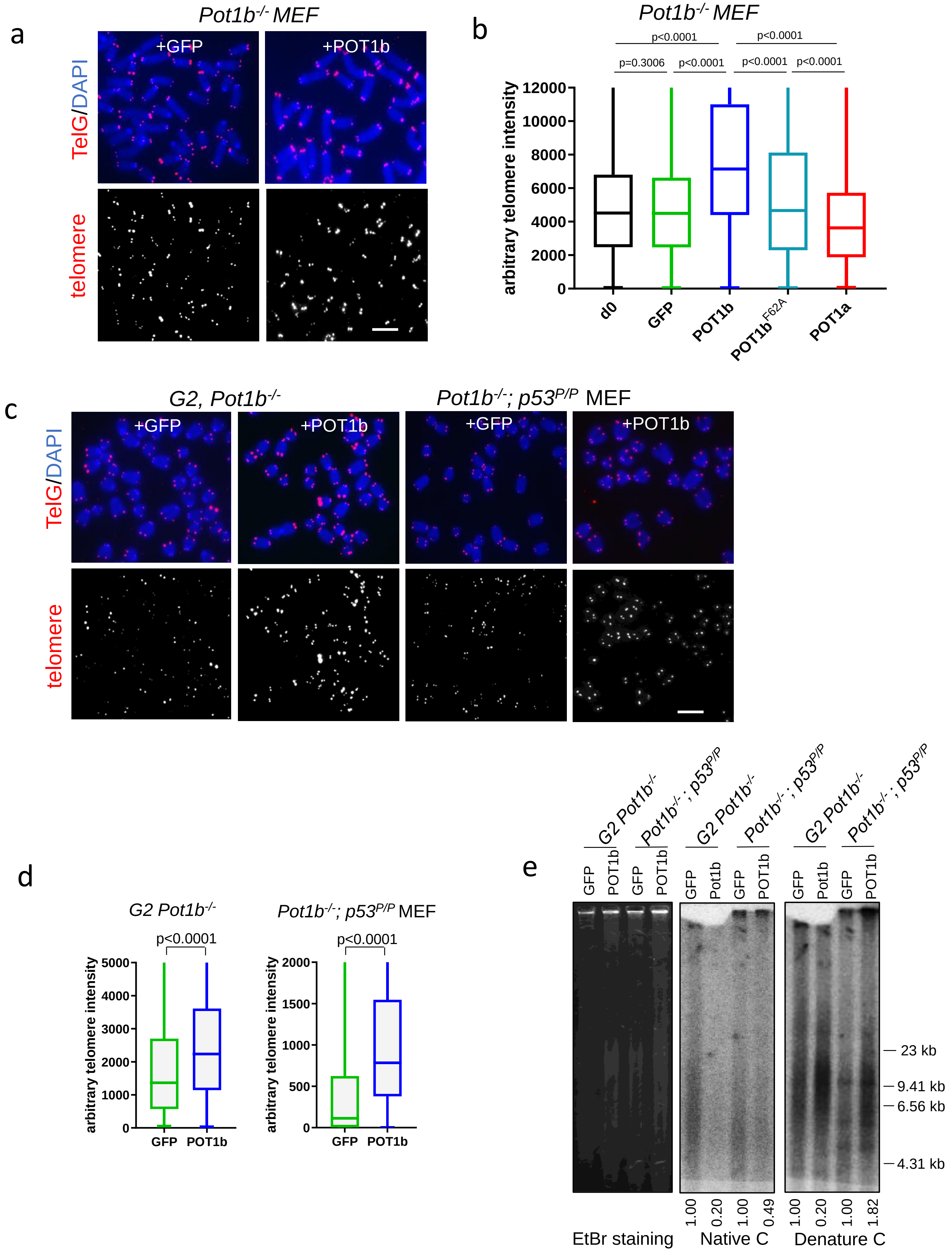

Supplementary Figure 2

Supplementary figure 2. Elongation of telomere by POT1b in other *Pot1b*$^{-/-}$ cell lines. a. PNA-FISH images of SV40 immortalized *Pot1b*$^{-/-}$ MEFs reconstituted with GFP or POT1b constructs. Scale bar: 5µm**. b.** Quantification of telomere intensity by Q-FISH in (**a**). A minimum of 30 metaphases were scored per sample. Box show the median with interquartile range (25% - 75%) from three independently repeated experiments. p-values are shown and generated from one-way ANOVA analysis followed by Tukey's multiple comparison. **c.** PNA-FISH images of SV40-immortalized *G2 Pot1b*$^{-/-}$ sarcoma cells and late passage (PD=125) *p53*$^{P/P}$*; Pot1b*$^{-/-}$ MEF after reconstitution with GFP or POT1b. *p53*$^{P/P}$: homozygous p53 R172P mutation. Scale bar: 5µm**. d**. Quantification of telomere intensity by Q-FISH in (C). A minimum of 30 metaphases were scored per sample. Data show the median with interquartile range (25% - 75%) from three experiments. p-values were generated from unpaired student's t-test. **e.** TRF Southern blot analysis of samples in (**c**). Ethidium bromide (EtBr) image shows that all samples contained equal amounts of genomic DNA. Molecular weight markers are indicted. Numbers indicate relative G-overhang and total telomere with telomere signals set to 1.0 for cells expressing GFP.

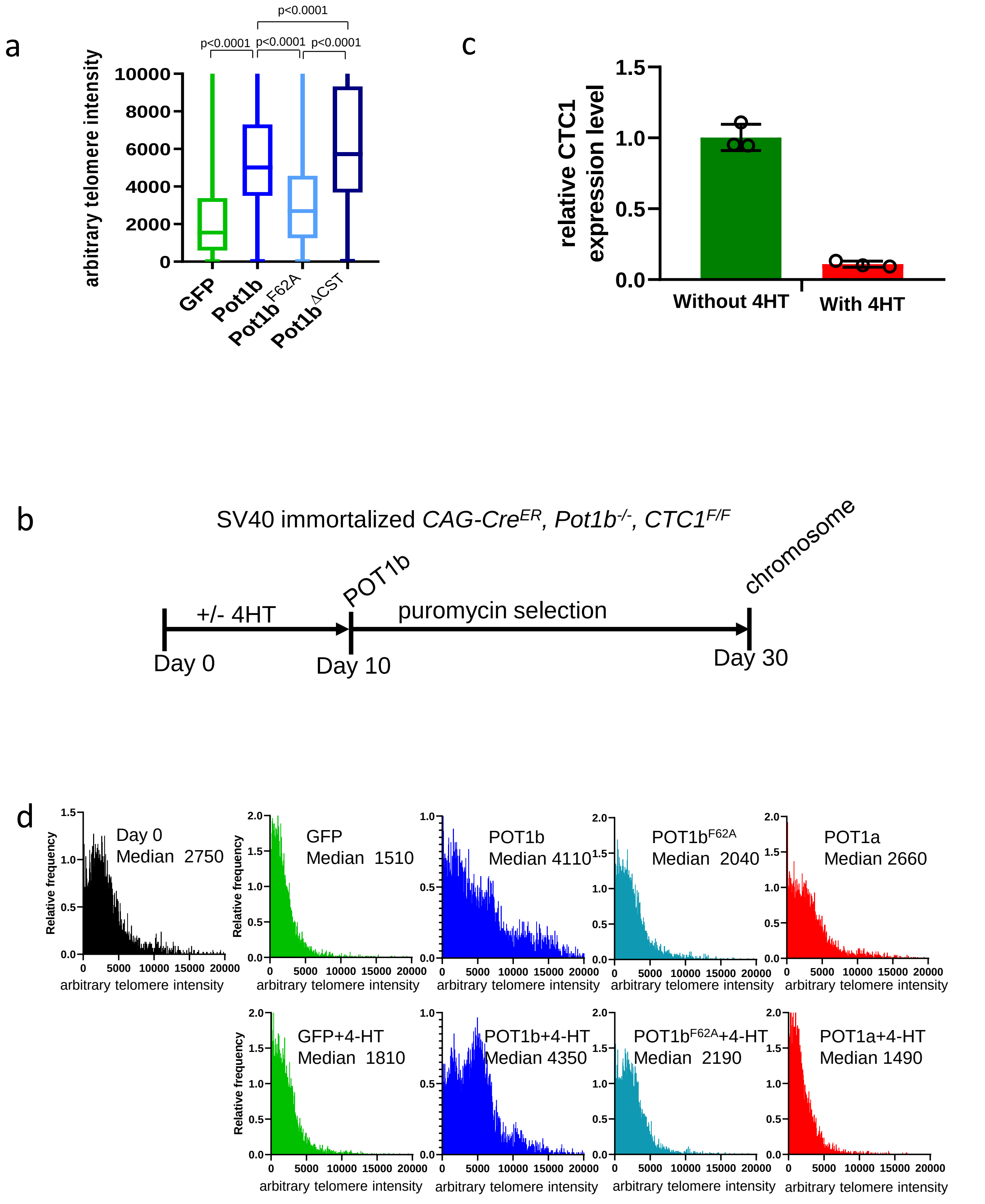

Supplementary Figure 3

Supplementary figure 3. POT1b-mediated telomere elongation occurs independent of CST. a. Quantification of telomere intensity in *G3 Pot1b*$^{-/-}$ sarcoma cells reconstituted with indicated POT1 (related to **Figure 2a**). A minimum of 30 metaphases were analyzed. Data show the median with interquartile range (25% - 75%) from three independent experiments. p-values are shown and generated from one-way ANOVA analysis followed by Tukey's multiple comparison. **b.** Schematic of treatment with 4-HT and reconstitution of POT1a or POT1b in *CAG-Cre*^{ER} *; Pot1b*$^{-/-}$*; CTC1*$^{F/F}$ MEFs. **c.** Real-time PCR to detect the depletion of CTC1 by 4-HT treatment at day 10. Data show mean ± s.d. from triplicate samples. **d**. Distribution of relative telomere lengths in *CAG-Cre*^{ER} *; Pot1b*$^{-/-}$*; CTC1*$^{F/F}$ MEFs reconstituted with indicated POT1a or POT1b wildtype or mutants +/- 4-HT (related to **Figures 2d-2e**). Telomeres from a minimum of 30 metaphases were scored in three independent experiments.

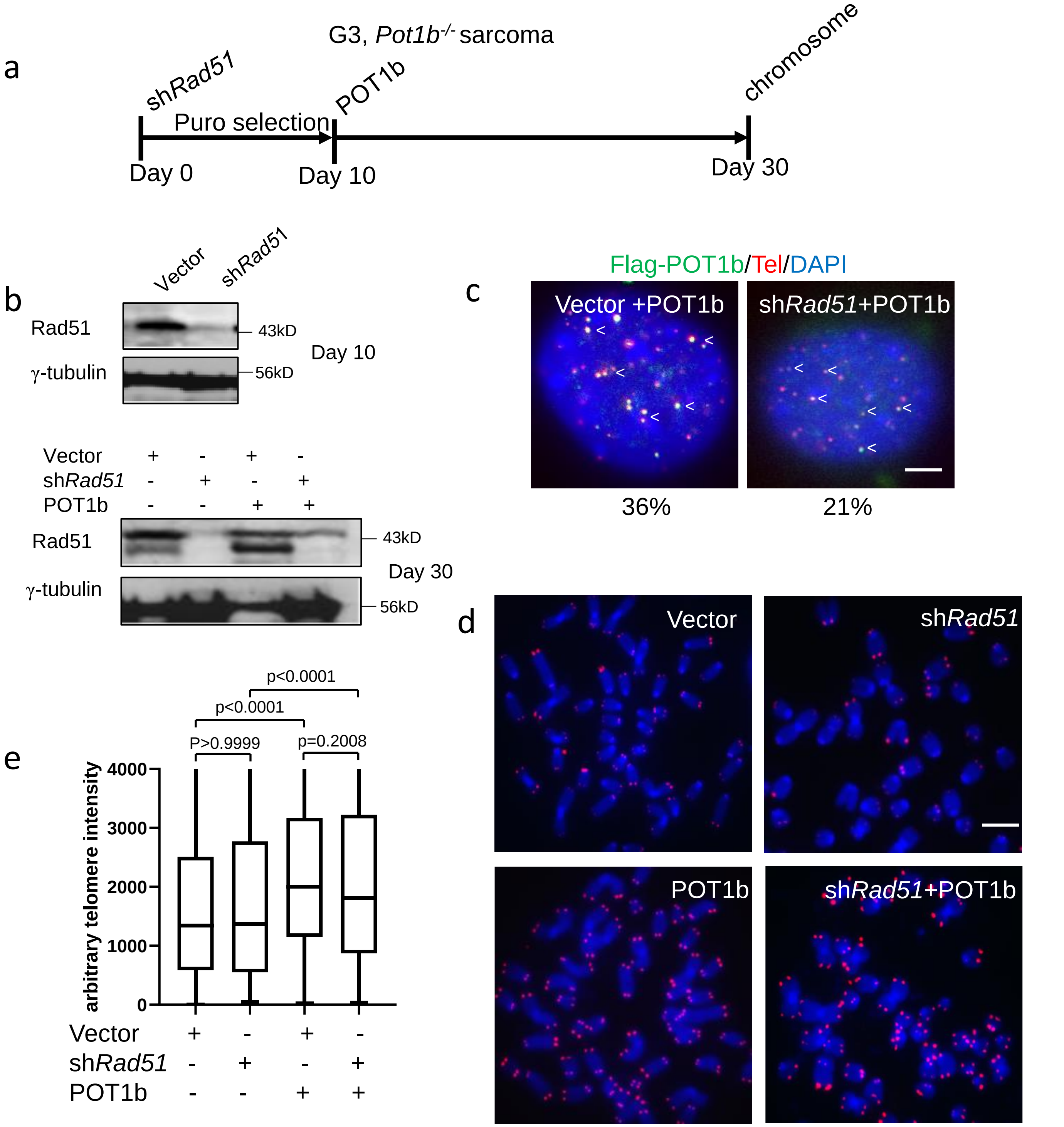

Supplementary figure 4

Supplementary figure 4. Telomere elongation by POT1b is not dependent on homologous recombination. a. Schematic of sh*Rad51* depleted *G3 Pot1b*$^{-/-}$ sarcomas reconstituted with POT1b. **b.** Western blot of cells treated with sh*Rad51* at days 10 and 30. γ-tubulin was used as loading control. **c.** Telomere-PNA immune-FISH was used to determine the localization (white arrows) of POT1b on telomere of *G3 Pot1b*$^{-/-}$ sarcomas at day 30. The numbers indicate the percentage of nuclei with greater than 5 POT1b foci on telomeres. Scale bar: 5µm. **d**. Telomere PNA-FISH of *G3 Pot1b*$^{-/-}$ sarcomas treated with sh*Rad51* and reconstituted with POT1b. Scale bar: 5µm. **e.** Quantification of arbitrary telomere intensity by Q-FISH in (**d**). At least 30 metaphases were scored in each treatment. Scale bar: 5µm. Data show the median with interquartile range (25% to 75%) from two independent experiments. p-values are shown and generated from one-way ANOVA analysis followed by Tukey's multiple comparison.

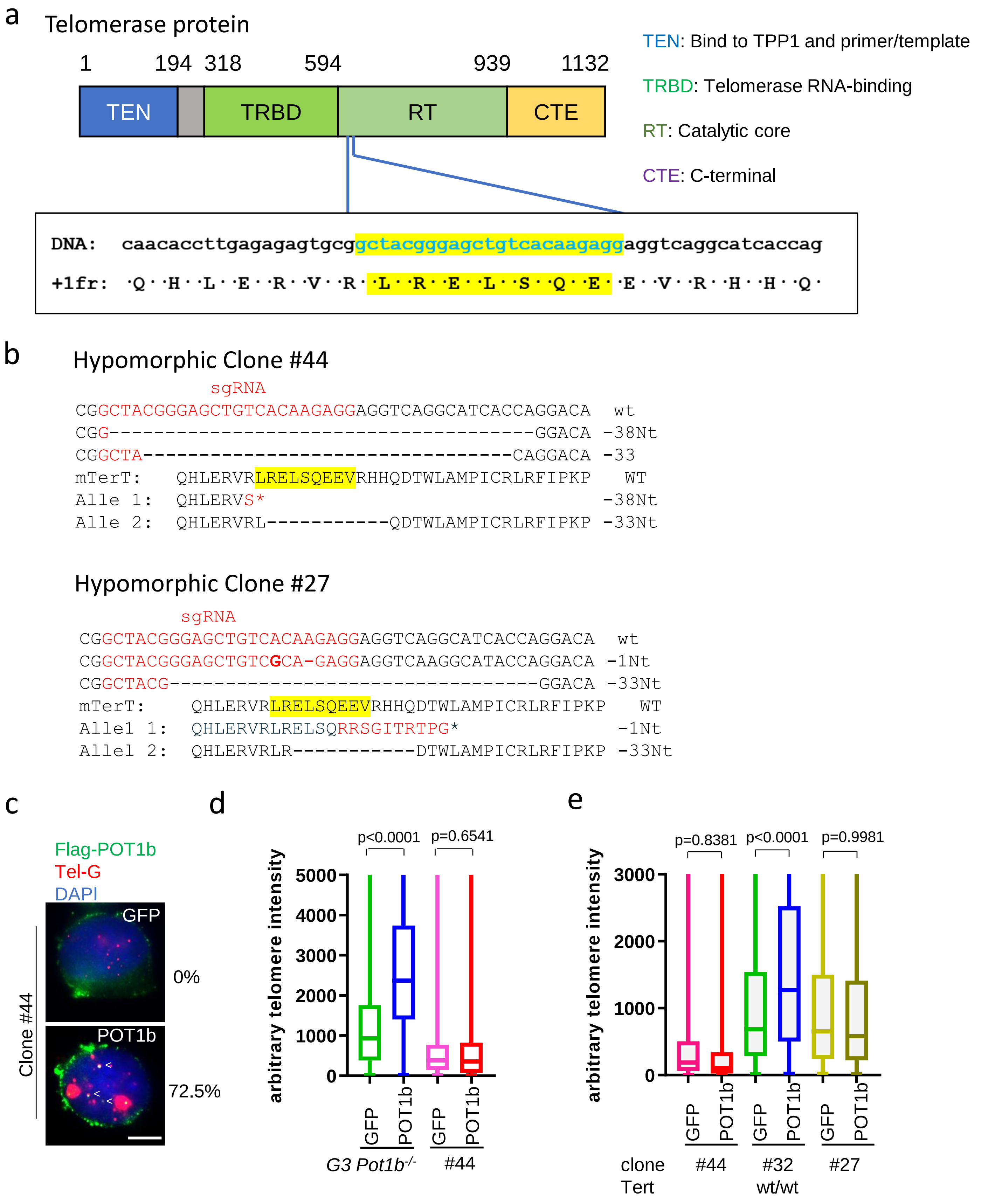

Supplementary figure 5.

Supplementary figure 5. CRISPR/Cas9 knockdown of telomerase in *G3 Pot1b$^{-/-}$* sarcoma cells. **a.** Schematic of the *mTert* gene, sgRNA sequence and targeting site (highlighted in yellow). **b.** mTert DNA sequences and predicted amino acid alignment surrounding the sgRNA targeting site in CRISPR/CAS9 deletion clones #44 and #27. All clones show one in-frame deletion *mTert* gene and one mutant *Tert* allele with a stop codon (*). **c.** Co-localization (white arrows) of Flag-POT1b (green) with telomeres (red) in clone #44. The numbers at the right site indicate the percentage of nuclei with more than 5 Pot1b foci co-localized at telomeres. Scale bar: 5µm**.** **d.** Quantification of telomere signals in *G3 Pot1b$^{-/-}$* parental cell and CRISPR/CAS9 targeted *Pot1b$^{-/-}$; Tert^{hypo}* clone #44 reconstituted with GFP or POT1b (related to **Figure 3c-3d**). At least 30 metaphases were scored per experiment. Data show the median with interquartile range (25% - 75%) from three independent experiments. p-values are shown and generated from unpaired student's t-test. **e.** Quantification of telomere signals in non-targeted clone #32, targeted *Pot1b$^{-/-}$; Tert^{hypo}* clones #44 and #27. Cells were reconstituted with either GFP or POT1b^{WT}. At least 30 metaphases were scored in each treatment. Data show the median with interquartile range (25% - 75%) from three independent experiments. p-values are shown and generated from unpaired student's t-test.

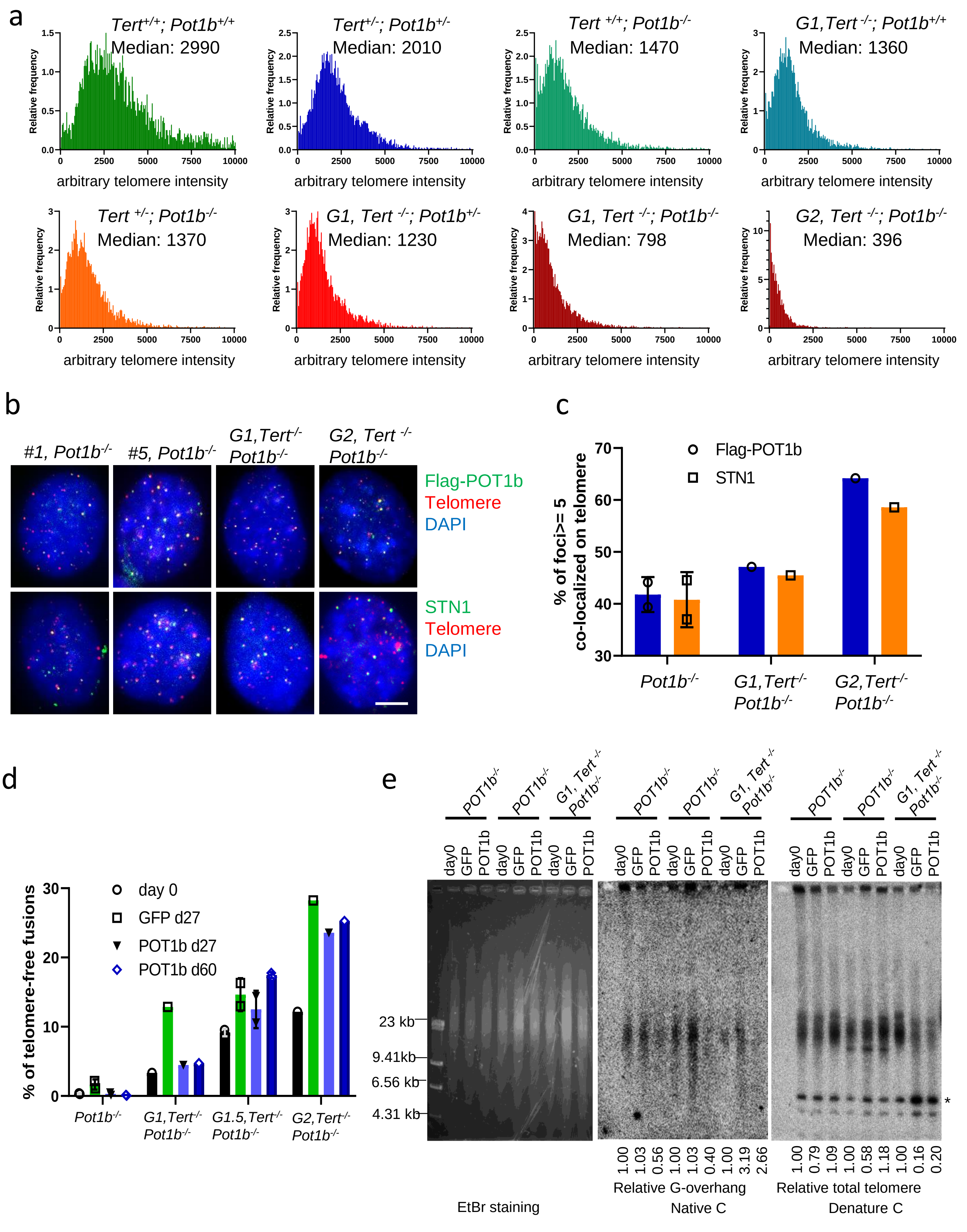

Supplementary figure 6

Supplementary figure 6. Accelerated telomere shortening in *Pot1b$^{-/-}$; Tert$^{-/-}$* cells. a. Distribution of relative telomere intensity measured by Q-FISH in primary MEFs with the indicated genotypes. A minimum of 40 metaphases were analyzed per genotype for Q-FISH in two independent experiments. More than 1500 chromosome were scored for telomere signals and data show the mean ± s.d. **b.** Images of co-localized POT1b foci and STN1 foci on telomeres in reconstituted *Pot1b$^{-/-}$*, G1 *Tert$^{-/-}$; Pot1b$^{-/-}$* and G2 *Tert$^{-/-}$; Pot1b$^{-/-}$* MEFs with POT1b. Scale bar: 5μm**.** **c.** Quantification of POT1b and STN1 foci on telomere in (**b**). At least 100 nuclei were scored each sample. The error bar was derived from the two *Pot1b$^{-/-}$* cell line. Data show mean ± s.d. **d.** Quantification of telomere-free end chromosome fusions in passaged MEFs reconstituted with GFP or POT1b and harvested at days 0, 27 or 60. At least 35 metaphases were scored in each sample. The error bar was derived from the two *Pot1b$^{-/-}$* cell line and two *G1.5, Tert $^{-/-}$; Pot1b$^{-/-}$* cell lines. Data show mean ± s.d. **e.** TRF Southern to detect the G-overhangs (native) and total telomere (denature) in *Pot1b$^{-/-}$*, G1 *Tert$^{-/-}$; Pot1b$^{-/-}$* MEFs reconstituted with GFP or POT1b for 27 days. Ethidium bromide (EtBr) image shows that all samples contained equal amounts of genomic DNA. Molecular weight markers are indicted. *: DNA band used for quantification. Numbers indicate relative G-overhang and total telomere signals, with telomere signals set to 1.0 for cells expressing GFP.

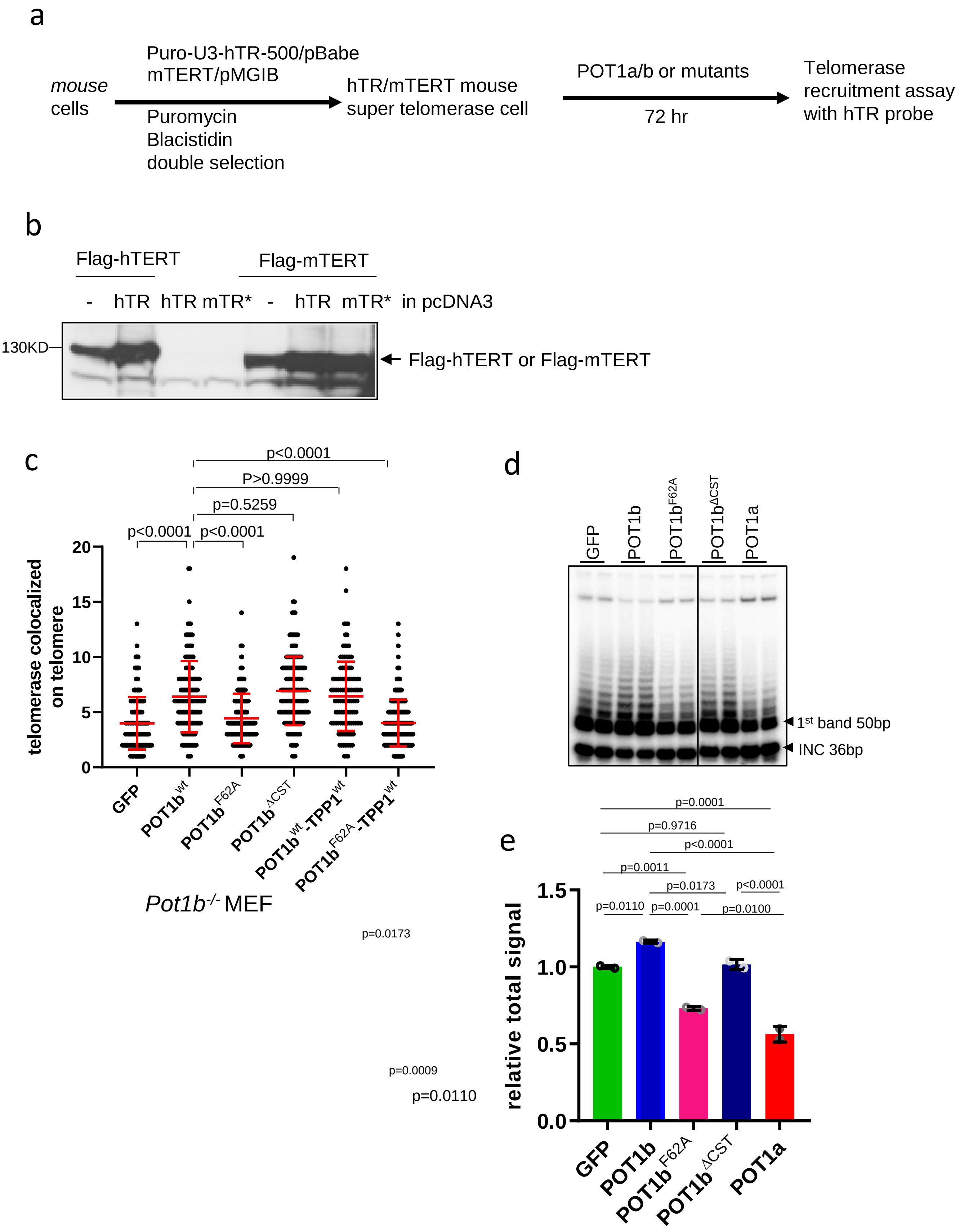

Supplementary figure 7

Supplementary figure 7. POT1b promotes telomerase recruitment to telomere. a. Schematic of telomerase recruitment assay in mouse cells expressing human telomerase RNA (hTR), mouse telomerase (mTERT), POT1a or POT1b WT and mutant constructs. **b.** Detection of Flag-tagged telomerase overexpressed in 293T cells in the presence or absence of human or mouse telomerase RNA (TR) by western blot. **c.** Telomerase recruitment assay in SV40 immortalized *Pot1b*$^{-/-}$ MEFs overexpressing POT1b^{WT}, POT1b mutants or tethered POT1b-TPP1 constructs. At least 500 nuclei were analyzed per genotype. Data show the mean ± s.d. from three independent experiments. p-values were shown and generated from one-way ANOVA analysis. **d.** TRAP assay showing telomerase activity in reconstituted *G3 Pot1b*$^{-/-}$ sarcoma cells expressing the indicated DNAs. Extracts from 2000 cells were used per lane. INC: internal control. **e.** Quantification of total TRAP PCR bands in (**d**). Data show mean ± s.d from duplicate samples. p-values were shown and generated from one-way ANOVA analysis followed by Tukey's multiple comparison.

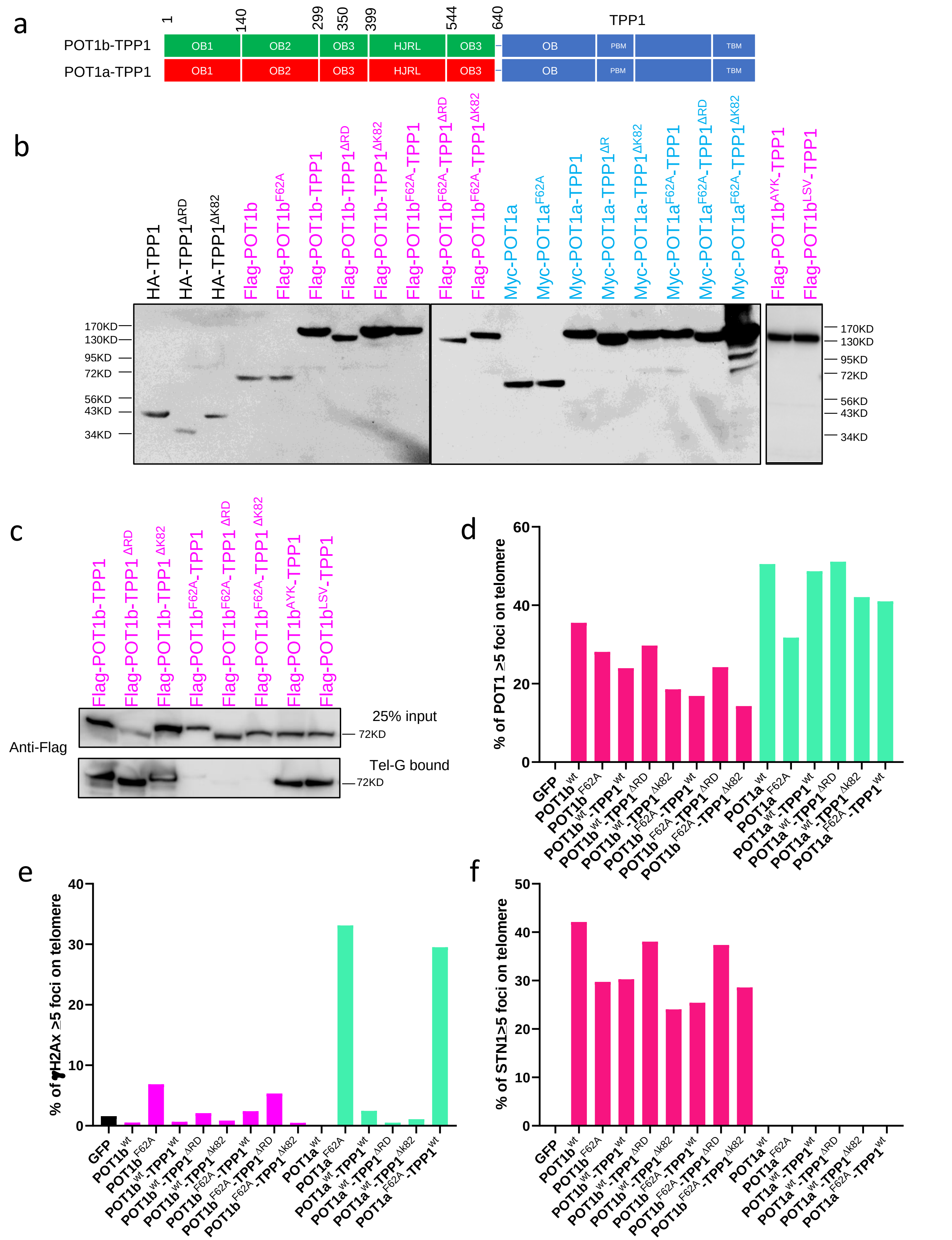

Supplementary figure 8

Supplementary figure 8. Characterization of tethered POT1-TPP1 DNA constructs. a. Schematic of POT1a and POT1b tethered to TPP1 with a flexible amino acid linker. Protein domains are indicated. **b.** Expression of POT1a, POT1b or tethered POT1-TPP1 constructs in 293T cells. Anti-epitope tag antibodies (anti-Flag, anti-HA and anti-Myc) were mixed together for Western blot analysis. **c**. Analysis of DNA binding activity of the tethered POT1b-TPP1 constructs on single-stranded Tel-G oligo [$(TTAGGG)_6$] in vitro. **d.** Quantification of co-localization of POT1a, POT1b or tethered POT1-TPP1 constructs on telomeres in *Pot1b*$^{-/-}$ cells infected with indicated POT1a or POT1b or tethered POT1-TPP1 constructs. **e**. Quantification of γ-H2AX foci on telomeres in *Pot1b*$^{-/-}$ cells expressing POT1a, POT1b or tethered POT1-TPP1 constructs. **f.** Quantification of STN1 foci on telomere in G0 *Pot1b*$^{-/-}$ MEFs expressing POT1a, POT1b or tethered POT1-TPP1 constructs.

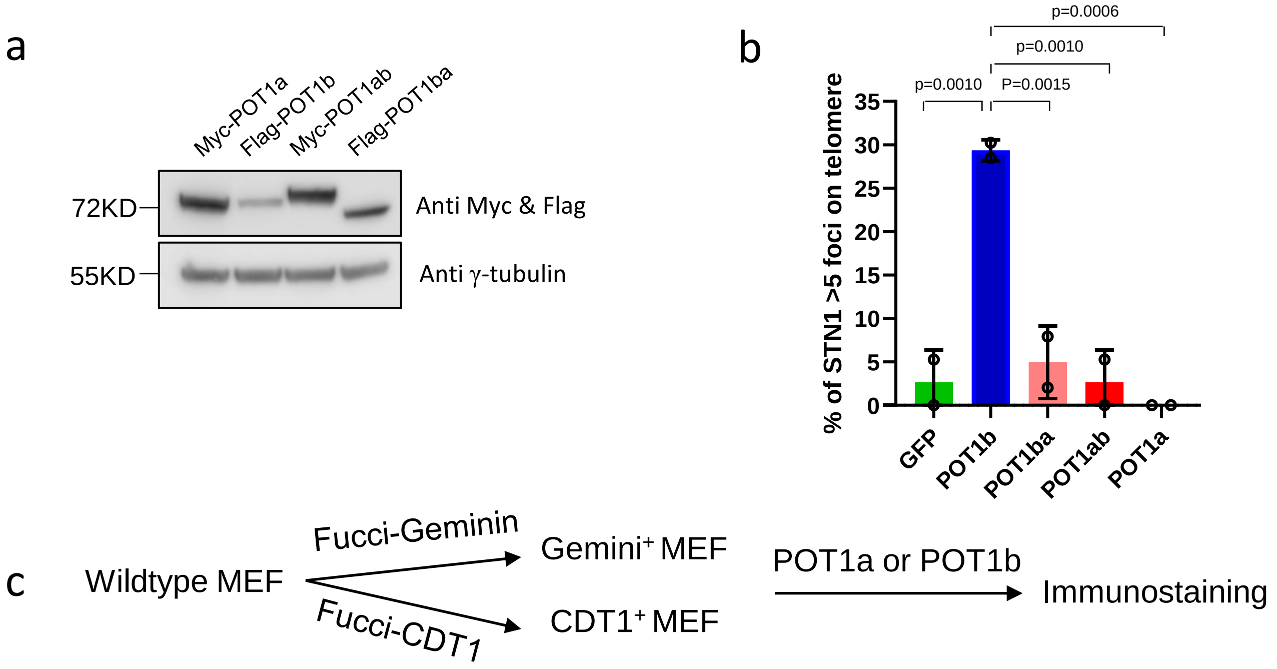

Supplementary figure 9

Supplementary figure 9. The POT1b C-terminal is required for telomerase recruitment to telomeres. **a.** Expression of Myc-POT1a, Flag-POT1b, Myc-POT1ab and Flag-POT1ba in 293T cells. **b.** Quantification of STN1 foci in G3 *Pot1b*$^{-/-}$ sarcomas reconstituted with GFP, POT1b^{WT}, POT1ab, POT1ba and POT1a^{WT}. Data show mean ± s.d. from two repeated experiments**.** p-values are shown and generated from one-way ANOVA analysis. **c**. Schematic of experiments to determine the influence of the cell cycle on the localization of POT1a or POT1b to telomeres in wild type MEFs expressing Fucci-Geminin or Fucci-CDT1.

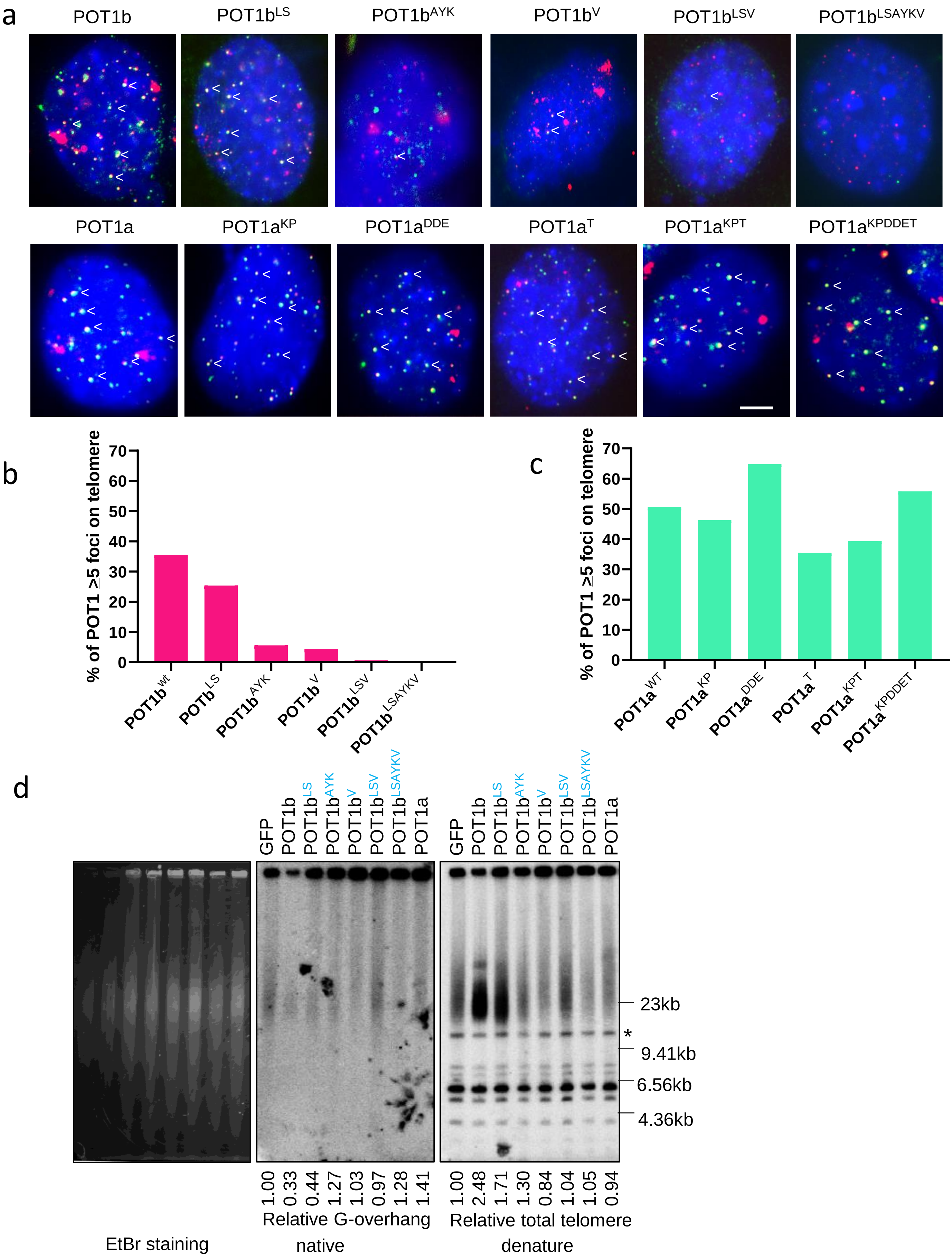

Supplementary figure 10

Supplementary figure 10. **Identification of unique amino acids in POT1b required for the elongation of telomere**. **a.** Immunostaining images showing that POT1a, POT1b and respective mutants localized (white arrows) to telomere in G3 *Pot1b*$^{-/-}$ sarcomas. Scale bar: 5μm**. b&c**. Quantification of POT1b positive foci (**b**) or POT1a positive foci (**c**) on telomere in (**a**). **d**. TRF southern blot to detect the G-overhang (native) and total telomere (denature) in G3 *Pot1b*$^{-/-}$ tumor cells reconstituted with POT1b^{WT} and POT1b mutants. Molecular weight markers are indicted. *: DNA band used for quantification. EtBr: Ethidium Bromide. Numbers indicate relative G-overhang and total telomere signals, with telomere signals set to 1.0 for cells expressing GFP. MW markers are indiated.

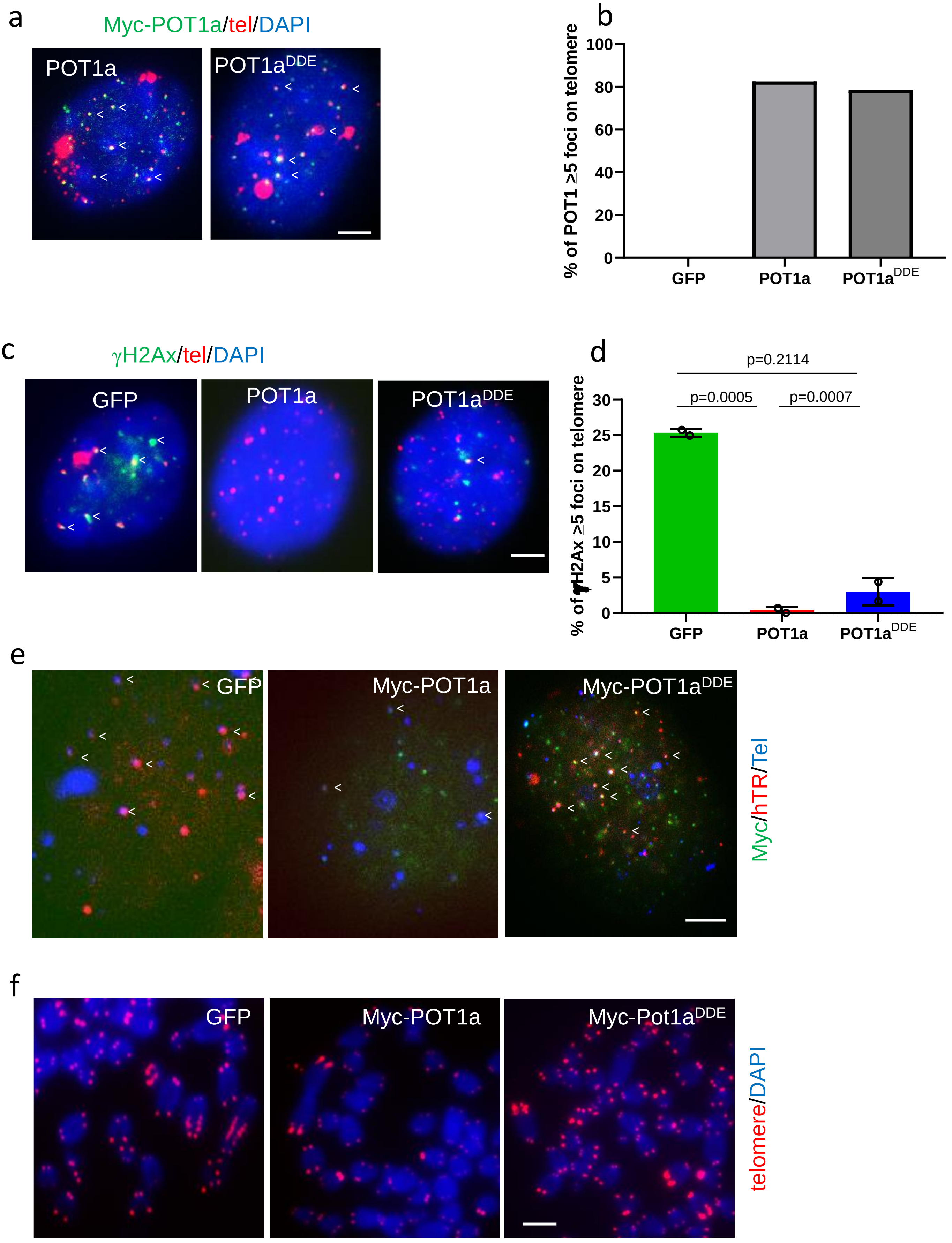

Supplementary figure 11

Supplementary figure 11. POT1a represses telomerase recruitment to telomeres and promotes telomere shortening. a. Immunostaining images showing POT1a^{WT} and POT1a^{DDE} localized (white arrows) to telomere in *MMTV-Cre; p53*$^{\Delta/\Delta}$*; Pot1a*$^{\Delta/\Delta}$ tumor cells. Scale bar: 5µm**.** **b.** Quantification of POT1a foci in (**a**). **c.** Immunostaining images to show γ-H2Ax localized (white arrows) to telomere in *MMTV-Cre; p53*$^{\Delta/\Delta}$*; Pot1a*$^{\Delta/\Delta}$ tumor cells expressing GFP, POT1a^{WT} or POT1a^{DDE}. Scale bar: 5µm**.** **d.** Quantification of γ-H2Ax foci in (**c**). Data show mean ± s.d from two independent experiments. p-values are shown and generated from one-way ANOVA analysis followed by Tukey's multiple comparison. **e.** (related to **Figure 6j**) Co-localization (white arrows) of hTR on telomere in *MMTV-Cre; p53*$^{\Delta/\Delta}$*; Pot1a*$^{\Delta/\Delta}$ tumor cells. Scale bar: 5µm**.** **f.** PNA-FISH images of *MMTV-Cre; p53*$^{\Delta/\Delta}$*; Pot1a*$^{\Delta/\Delta}$ tumor cells reconstituted with GFP, POT1a^{WT} or POT1a^{DDE} (related to **Figure 6k**). Scale bar: 5µm**.**

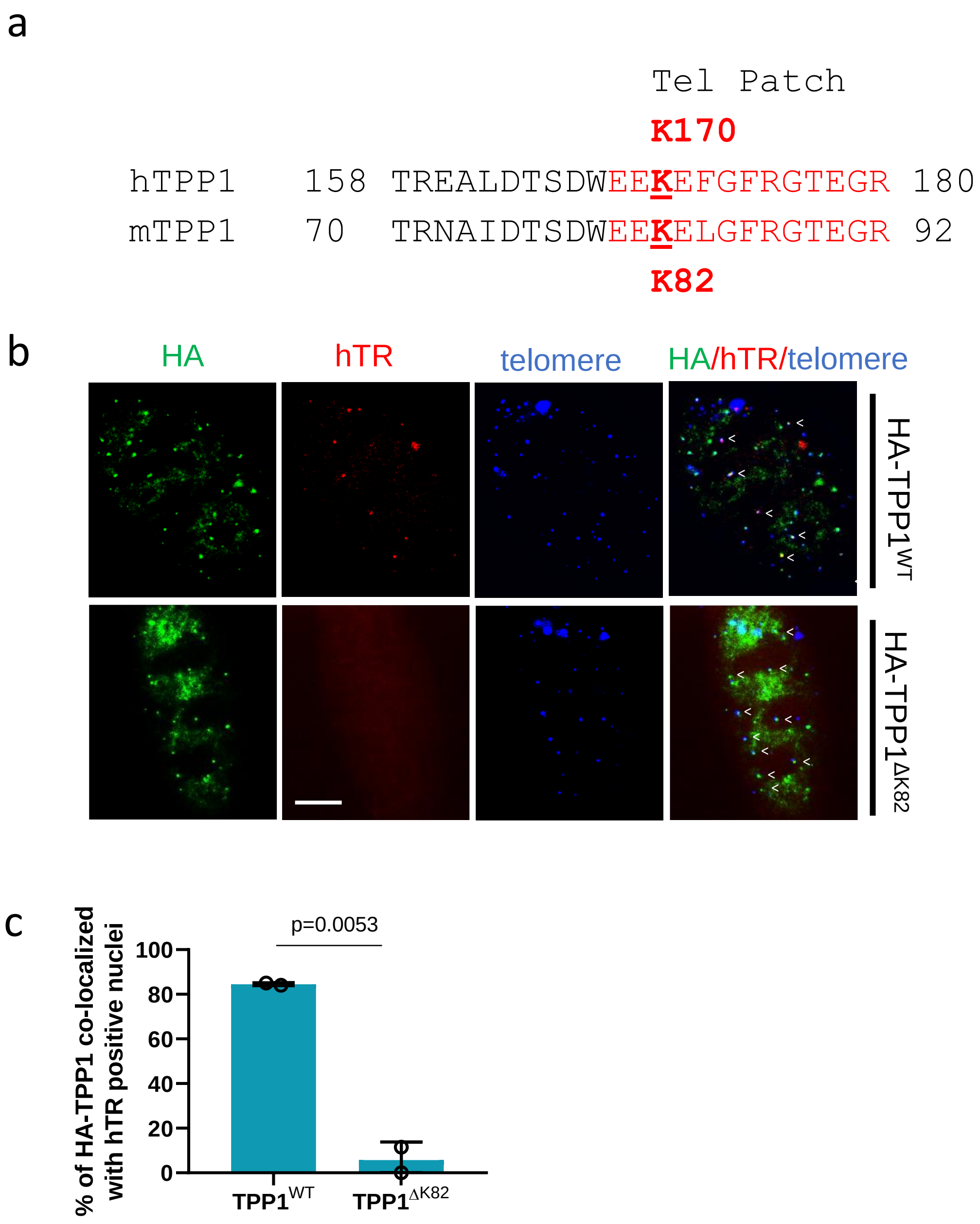

Supplementary figure 12

Supplementary figure 12. mTPP1$^{\Delta K82}$ impairs the recruitment of telomerase to telomeres. a. Alignment of amino acid sequence of human and mouse TPP1 TEL patch region. **b.** Examples of colocalization (white arrows) of hTR with wildtype mTPP1 or mTPP1$_{\Delta}^{K82}$ on telomere in *Pot1b*$^{-/-}$ cells. Scale bar: 5µm**.** **c.** Quantification of co-localization of hTR with mTPP1 or mTPP1$_{\Delta}^{K82}$ on telomeres. Nuclei containing more than 2 hTR foci co-localized with TPP1 was scored as positives. Error bars are derived from two independent experiments. p-values are shown and generated from unpaired student's t-test.

Supplementary table 1: primer list

	primer name	primer sequence
genotyping	Pot1a-EcoRI_21196	CTC GAA TTC CAT CTC CTC CCA GTA CTC TCT CAG
	Pot1a-Confirm_20560	GGA ACT GGT ACG TAT CAG TGT GTG TGG
	Pot1a-BamHI_16490	ACA CGG ATC CTG AGC CAT AAA CAT GCC ACA CAA AGG
	Pot1b-WT-A	TGTCTGTGGTGTTGTGAAGTTCT
	Pot1b-WT-B	TTCCTCTCCCAACACACCTT
	Pot1b-delta-A	GGGATGGTTCTGTGCAAGTG
	Cre-up	TCCAATTTACTGACCGTACACCAA
	Cre-down	CCTGATCCTGGCAATTTCGGCTA
	P53-loxFWB	AAG GGG TAT GAG GGA CAA GG
	p53-loxRVB	GAA GAC AGA AAA GGG GAG GG
	p53-1F	CAC AAA AAC AGG TTA AAC CCA G
	CTC1-sp2	GATAGTGATACAGATAATAGATGGAAC
	CTC1-sp4	CGTGCATATCTATGTACCACTTGC
	CTC1-sp6	GTAAAGGCAGGCAAAGAGGATGAT
RT-PCR	CTC1-RT-F1	ATGACACTTGGTGAATTAGCCC
	CTC1-RT-R1	AGGAAAGGTGAATCCGGGTCT
Pot1b point mutation	Pot1b-LS-F	agcaaagctaagatcatacctgcccagaagactttctcagtctgttaaacttt
	Pot1b-LS-R	aaagtttaacagactgagaaagtcttctgggcaggtatgatcttagctttgct
	Pot1b-AYK-F	cagactgcaagctgcaagctacaccattgtattactcaaaggtctggaccactgaag
	Pot1b-AYK-R	cttcagtggtccagacctttgagtaatacaatggtgtagcttgcagcttgcagtctg
	Pot1b-T551V-F	cctctacagtatgtgtttgttatggttttcatgtttgatgatggaaccgg
	Pot1b-T551V-R	ccggttccatcatcaaacatgaaaaccataacaaacacatactgtagagg
Pot1a point mutation	Pot1a-KP-F	ccggttccatcatcaaacatgaaaaccataacaaacacatactgtagagg
	Pot1a-KP-R	gagaagtttaacagactggggaagtctcctgggcttataggatctcagtttggcg
	pot1a-DDE-F	aagctccagacatcaagctgaaagacacatcattgtatgactcagaagtctggaccactgaagac
	pot1a-DDE-R	gtcttcagtggtccagacttctgagtcatacaatgatgtgtctttcagcttgatgtctggagctt
	Pot1a-V551T-F	ctgcagtatgtgtttgttatgactttcactctggatgacgggac
	Pot1a-V551T-R	gtcccgtcatccagagtgaaagtcataacaaacacatactgcag
mTPP1$^{\Delta K82}$ point mutation	mTPP1delK82-F	5'-cgactgggaggaggagctcggattcc-3'
	mTPP1delK82-R	5'-ggaatccgagctcctcctcccagtcg-3'
tethered mPot1-mTPP1 linker	mPot1a-mTPP1-F	actacagttgcagaaaatgttgtcGGAGGATCTGGTGGAAGTGGTGGAtccgattcagggttgctggcccta
	mPot1a-mTPP1-R	tagggccagcaaccctgaatcggaTCCACCACTTCCACCAGATCCTCCgacaacattttctgcaactgtagt
	mPot1b-mTPP1-F	accatgattgcagaagacatcatcGGAGGATCTGGTGGAAGTGGTGGAtccgattcagggttgctggcccta
	mPot1b-mTPP1-R	tagggccagcaaccctgaatcggaTCCACCACTTCCACCAGATCCTCCgatgatgtcttctgcaatcatggt
CRISPR/Cas9 knockdown mTert	SgmTert-3F	CACCGCTACGGGAGCTGTCACAAG
	SgmTert-3R	AAACCTTGTGACAGCTCCCGTAGC
	PCR-F1	CTGCATGCTCCTGTCATAACTC
	PCR-R	GACTCAACCATCAGTACAGGGG
	PCR-F2	TTCGTGTCATGAACCCTGTCCAGAC
human telomerase RNA probes	hTR_FISH_1	5'-/5Cy5/GCT GAC ATT TTT TGT TTG CTC TAG AAT GAA CGG TGG AAG GCG GCA GGC CGA GGC TT
	hTR_FISH_2	5'-/5Cy5/CTC CGT TCC TCT TCC TGC GGC CTG AAA GGC CTG AAC CTC GCC CTC GCC CCC GAG AG
	hTR_FISH_3	5'-/5Cy5/ATG TGT GAG CCG AGT CCT GGG TGC ACG TCC CAC AGC TCA GGG AAT CGC GCC GCG CGC
