# Supplementary material for: Distinct functions of POT1 proteins contribute to the regulation of telomerase recruitment to telomeres
Source: Nat Commun. 2021 Sep 17;12:5514. doi: 10.1038/s41467-021-25799-7 (PMC8448735; doi:10.1038/s41467-021-25799-7)
Supplement: Supplementary file 3 — Reporting Summary [file 41467_2021_25799_MOESM3_ESM.pdf]

nature portfolio

Corresponding author(s): Sandy Chang

Last updated by author(s): 08/06/2021

Reporting Summary

Nature Portfolio wishes to improve the reproducibility of the work that we publish. This form provides structure for consistency and transparency in reporting. For further information on Nature Portfolio policies, see our Editorial Policies and the Editorial Policy Checklist.

Statistics

For all statistical analyses, confirm that the following items are present in the figure legend, table legend, main text, or Methods section.

n/a	Confirmed	
☐	☒	The exact sample size (*n*) for each experimental group/condition, given as a discrete number and unit of measurement
☐	☒	A statement on whether measurements were taken from distinct samples or whether the same sample was measured repeatedly
☐	☒	The statistical test(s) used AND whether they are one- or two-sided<br>*Only common tests should be described solely by name; describe more complex techniques in the Methods section.*
☐	☒	A description of all covariates tested
☐	☒	A description of any assumptions or corrections, such as tests of normality and adjustment for multiple comparisons
☐	☒	A full description of the statistical parameters including central tendency (e.g. means) or other basic estimates (e.g. regression coefficient) AND variation (e.g. standard deviation) or associated estimates of uncertainty (e.g. confidence intervals)
☐	☒	For null hypothesis testing, the test statistic (e.g. *F*, *t*, *r*) with confidence intervals, effect sizes, degrees of freedom and *P* value noted<br>*Give P values as exact values whenever suitable.*
☒	☐	For Bayesian analysis, information on the choice of priors and Markov chain Monte Carlo settings
☐	☒	For hierarchical and complex designs, identification of the appropriate level for tests and full reporting of outcomes
☒	☐	Estimates of effect sizes (e.g. Cohen's *d*, Pearson's *r*), indicating how they were calculated

Our web collection on statistics for biologists contains articles on many of the points above.

Software and code

Policy information about availability of computer code

Data collection	Software NIS-Elements BR (Nikon) v3.22.11
Data analysis	ImageJ v1.51k (NIH)<br>GraphPad Prism v8.0

For manuscripts utilizing custom algorithms or software that are central to the research but not yet described in published literature, software must be made available to editors and reviewers. We strongly encourage code deposition in a community repository (e.g. GitHub). See the Nature Portfolio guidelines for submitting code & software for further information.

Data

Policy information about availability of data

All manuscripts must include a data availability statement. This statement should provide the following information, where applicable:

- Accession codes, unique identifiers, or web links for publicly available datasets
- A description of any restrictions on data availability
- For clinical datasets or third party data, please ensure that the statement adheres to our policy

The authors declare that all data supporting the findings of this study are available within the paper and its supplementary information files. Source data are provided with this paper.

Field-specific reporting

Please select the one below that is the best fit for your research. If you are not sure, read the appropriate sections before making your selection.

☒ Life sciences ☐ Behavioural & social sciences ☐ Ecological, evolutionary & environmental sciences

For a reference copy of the document with all sections, see nature.com/documents/nr-reporting-summary-flat.pdf

Life sciences study design

All studies must disclose on these points even when the disclosure is negative.

Sample size	No statistical tests were performed to predetermine the sample sizes. Sample sizes were chosen based on previous publications and a sufficient number of replicates was performed to allow calculations of statistical significance.
Data exclusions	No data were excluded from the analysis.
Replication	All the experiments were successfully replicated either two or three times. Details on the number of replicates for each experiment can be found in the associated figure legend. When possible, alternative methods were used to confirm the results.
Randomization	Allocation of samples into the experimental groups was random and the experiments were carried out in parallel.
Blinding	Investigators were blinded to group allocation during both data collection and data analysis for all the experiments involving fluorescence microscopy. Data interpretation of other experiments was based on appropriate controls.

Reporting for specific materials, systems and methods

We require information from authors about some types of materials, experimental systems and methods used in many studies. Here, indicate whether each material, system or method listed is relevant to your study. If you are not sure if a list item applies to your research, read the appropriate section before selecting a response.

Materials & experimental systems

n/a	Involved in the study	
☐	☒	Antibodies
☐	☒	Eukaryotic cell lines
☒	☐	Palaeontology and archaeology
☐	☒	Animals and other organisms
☒	☐	Human research participants
☒	☐	Clinical data
☒	☐	Dual use research of concern

Methods

n/a	Involved in the study	
☒	☐	ChIP-seq
☒	☐	Flow cytometry
☒	☐	MRI-based neuroimaging

Antibodies

Antibodies used	Primary antibodies<br>Phospho-Histone H2AX (S139) mouse monoclonal antibody (Millipore), cat #05-636, clone JBW301, lot 3108494. Dilution 1:1000<br>RAD51 rabbit polyclonal antibody (Santa Cruz), cat #sc-8349, clone H-92, lot J2014. Dilution 1:1000<br>STN1 rabbit polyclonal antibody (Chang lab-made using full-length mouse STN1 peptide), Dilution 1:1000<br>FLAG M2 mouse monoclonal antibody (Sigma), cat #F3165, lot SLBT6752. Dilution 1:5000<br>HA mouse monoclonal antibody (Sigma), cat#H3663, lot 038m4810v, Dilution 1:5000<br>Myc mouse monoclonal antibody (Millipore), cat #05-724, clone 4A6, lot 3095953. Dilution 1:5000<br>Gamma-tubulin mouse monoclonal antibody (Sigma), cat# T6557, clone GTU-88, lot 049M4786V. Dilution 1:5000<br>Secondary antibodies:<br>Peroxidase-linked anti-mouse IgG (Amersham), cat #NXA931V, lot 16964893. Dilution 1:5000<br>Peroxidase-linked anti-rabbit IgG (Amersham), cat #NA934V, lot 16991099. Dilution 1:5000<br>Alexa Fluor 488 goat anti-mouse (Invitrogen), cat #A11001, lot 2140660. Dilution 1:1000
Validation	Phospho-Histone H2AX (S139) (05-636): validation can be found in the manifacturer's website https://www.emdmillipore.com/US/en/product/Anti-phospho-Histone-H2A.X-Ser139-Antibody-clone-JBW301,MM_NF-05-636. Used in several studies for immunostaining experiments: d'Adda di Fagagna F., et al. (2003) Nature: 426:194-8. Lin J.R., et al. (2015) Nat. Commun. 6:8390. Rai R., et al. (2019) Cell Rep. 29(11):3708-3725.e5.<br>RAD51 antibody (sc-8349): https://www.scbt.com/p/rad51-antibody-h-92. Validated for immunostaining in human and mouse cell lines. Nicolae C.M., et al. (2015) Nucleic acids research. 43: 3143-53. Rai R., et al. (2016) Nature communications. 7: 10881. Pathania<br>3<br>nature portfolio | reporting summary March 2021<br>S., et al. (2014) Nature Commun. 5: 5496.

STN1 rabbit polyclonal antibody was made in Chang lab using bacteria expressed full-length mouse STN1 peptide. Validated for immunostaining in mouse cells and Western blot. Gu P (2012) EMBO J 31, 2309-2321
FLAG M2 antibody (F3165): https://www.sigmaaldrich.com/catalog/product/sigma/f3165?lang=en&region=US. Validated for Western blot and immunostaining for the detection of FLAG tag. Rai R., et al. (2019) Cell Rep. 29(11):3708-3725.e5. Sriramachandran A. M., et al. (2019) Nat Commun. 15;10(1):3678. Doyle S. L., et al. (2012) Nat Commun. 28;3:707.
HA antibody (H3663):https://www.https://www.sigmaaldrich.com/US/en/product/sigma/h3663?context=product# Validated for use in CHIP, IC, IP, WB for the detection of HA Tag. Chen C. et al (2017) Nat Commun 8, 14929, Markus H et al (2020) Cell, 181(2).
Myc antibody (05-724): https://www.emdmillipore.com/US/en/product/Anti-Myc-Tag-Antibody-clone-4A6,MM_NF-05-724. Validated for use in ChIP, IC, IF, IP, WB for the detection of Myc Tag. Rai R., et al. (2019) Cell Rep. 29(11):3708-3725.e5. Zhang S., et al. (2015) Nat Neurosci. 18(3):386-92.
Gamma-tubulin antibody (T6557): https://www.sigmaaldrich.com/catalog/product/sigma/t6557?lang=en&region=US. Validated for Western blot and immunostaining in human cell lines. Rai R., et al. (2019) Cell Rep. 29(11):3708-3725.e5. Rai R., et al. (2017) Mol Cell 65(5): 801–817.e4. Rai R., et al. (2016) Nature Commun. 7: 10881.

Eukaryotic cell lines

Policy information about cell lines

Cell line source(s)	293T: ATCC (CRL-3216). All MEFs and sarcoma cell lines were generated in Chang lab
Authentication	These cell lines are routinely used in our lab and we constantly monitor their morphology.
Mycoplasma contamination	All cell lines tested negative for mycoplasma contamination by PCR.
Commonly misidentified lines (See ICLAC register)	No commonly misidentified cell lines were used in this study.

Animals and other organisms

Policy information about studies involving animals; ARRIVE guidelines recommended for reporting animal research

Laboratory animals

The species being used is Mus musculus. The parental strains utilized in this research proposal to generate mouse embryo fibroblasts and derivative sarcomas are POT1aF/F; POT1b-/-; p53F/F conditional knockout mice in a C57/B6 genetic background. The NSG (NOD/LtSscidIL2Rynull) immunocompromised mice was used as recipients. For generation of compound mouse cohorts, care was taken to ensure that there are no mating alleles are on the same chromosomes.
Mouse husbandry: The animal care and use program at Yale University maintains full accreditation from the Association for Assessment and Accreditation of Laboratory Animal Care (AAALAC) and complies with U.S. Animal Welfare Regulations, the National Research Council (NRC) Guide for the Care and Use of Laboratory Animals, and Public Health Service Policy on Humane Care and Use of Laboratory Animals. Yale University has an approved Animal Welfare Assurance (#A3230-01) on file with the NIH Office for Protection from Research Risks. The Assurance was approved May 16, 2007. My mouse personnel have multiple years of experience working with mice. Our veterinarian Dr. Peter Smith is always available should health concerns arise. My animal protocols have been approved by the animal care and use program at Yale University (2010-11358) for three years and renewed annually. Animal models and their derivative cell lines such as the ones described in this proposal have made a major impact on our understanding of fundamental biological processes, including molecular mechanisms governing genome stability. The ability to manipulate a specific gene in a controlled genetic background is extremely important to understand the impact of its deletion on biochemical processes.
Veterinary care and procedures regarding discomfort, distress, pain and injury: The animals are housed in a germ-free facility and maintained by technicians at our Animal Facilities Center. Veterinarians make weekly rounds to identify sick animals. These animals are either treated with appropriate measures or euthanized. All efforts will be made to ensure that discomfort, distress, pain and injury will be limited to that which is unavoidable in the conduct of scientifically sound research. Mice of both sexes will be monitored daily for the development of morbidity and/or tumors and will be sacrificed before symptoms become debilitating. Morbidity will be evaluated by physical appearance, lethargy/weakness, body weight, and dyspnea. Tumor size will not exceed 1 cm in maximum dimension where visible.
Mouse were kept at 12h/12h light/dark cycle. The animal room is maintained at ambient temperature range of room temperature. Maximal number of mouse per cage is 5. Male and female mice were maintained in separate cages before set up mating cage.
Method of euthanasia: Animals will be euthanized by CO2, an approved method consistent with recommendation of the Panel on Euthanasia of the American Veterinary Medical Association.

Wild animals	No wild animals were used
Field-collected samples	Our studies do not involved field-collected samples
Ethics oversight	Our study protocol was approved by IACUC of Yale University

Note that full information on the approval of the study protocol must also be provided in the manuscript.
